# Estimation of the biogas production rate, a chemometrical approach

**DOI:** 10.1186/1758-2946-6-S1-P26

**Published:** 2014-03-11

**Authors:** Tetyana Beltramo, Susanne Theuerl, Michael Klocke, Bernd Hitzmann

**Affiliations:** 1 Department of Process Analytics and Cereal Technology, Institute of Food Science and Biotechnology, University of Hohenheim, Stuttgart, 70599, Germany; 2 Leibniz Institute of Agricultural Engineering, Potsdam-Bornim, 14469, Germany

## 

Biogas production rate is an important criterion for the entire biogas production process. In the present study the biogas production rate was evaluated using more than 30 process variables measured at an agricultural biogas plant in Germany during two months. The measured variables include the chemical measurements (such as pH, dried matter, amount of organic acids), energy supply specifications, temperature level and substrate ingredients. The prediction of the biogas production rate was done using chemometric methods. The results of the different methods were compared and the most accurate method was identified. Here the cross-validated prediction error (RMSECV) computed using leave-one-out method was less than 5 percent for both PCR and PLSR models (less than 190 m^3^/d), while the calculated correlation coefficient (r^2^) for PLSR reached 0,85 and 0,75 for PCR. For better prediction accuracy a metaheuristic search of the process relevant variables was performed. Here the Ant Colony Optimisation (ACO) improved the prediction performance of PLSR, decreasing the RMSECV to less than 2 percent (95 m^3^/d) while increasing the r^2^ to 0,98. These are promising results, which prove the feasibility of using this evaluation methodology for monitoring in biogas production processes.

